# Medical Items

**Published:** 1912-03

**Authors:** 


### MEDICAL ITEMS

### DR. J. CHESTON KING HEADS FLORIDA SANITORIUM.

Plans for the establishment of the Homewood Sanitarium at Sulphur Springs by the Homewood Sanitarium company, articles for the incorporation of which are about to be filed with Governor Gilchrist at a capitalization of $500,000, promise for Tampa and south Florida one of the biggest drawing cards this section of the state has ever seen.

Organized with Dr. J. Cheston King, of Atlanta, at its head, and amply backed by the representative local business men, the Homewood Sanitarium Company is laying careful plans for the carrying out of its purpose—the establishment at Sulphur Springs of a sanitarium that shall be ranked among the foremost in the county.

